# Connecticut

**Published:** 1897-04

**Authors:** 


*Connecticut.* During 1896 some 6300 cattle were examined for tuberculosis and 897 were condemned and destroyed. The average value of animals condemned was $24.47. The total cost of the entire work for the year, including commissions, fees, animals condemned, veterinary services, etc., was $31,639.63. Of this amount, $3497.69 was paid as professional fees to veterinarians.

